# SOCIAL NETWORK AND CAREGIVING EXPERIENCE AMONG SPOUSE AND ADULT-CHILD DEMENTIA CAREGIVERS: A MEDIATION ANALYSIS

**DOI:** 10.1093/geroni/igad104.1869

**Published:** 2023-12-21

## Abstract

Evidence on differences in social network and their roles in associations between types of family caregivers and caregiving experience is limited. Based on the stress process model (SPM), we aimed to explore the different levels and interactions of social network and caregiving experience among spouses and adult-child caregivers. A questionnaire-based survey was conducted on a total of 146 dementia family caregivers (78 adult-child, and 68 spouses) in China. Data collection comprised four sections: (a) care-related stressors, (b) caregivers’ context, (c) social network using Lubben social network scale, and caregiving experience using short-form Zarit burden interview, and nine-item positive aspects of caregiving (PAC) scale. Linear regression, mediation model analysis, and interactive analysis were performed to explore associations between variables. Spouses experienced lower social network (β= -0.294, P=0.008) and higher PAC (β= 0.234, P=0.003) than adult-child caregivers, whereas no significant difference was found in caregiver burden between two groups. Mediation analysis suggested that associations between caregiver type and caregiver burden were indirect-only mediation effects by social network(β=0.140, 95%CI=0.066 to 0.228). The mediation effect of social network on caregiver type and PAC was the suppressing effect, and the interaction effect was significant (P for interaction =0.025). A higher social network was associated with higher positive aspects of caregiving among the spouse subgroup (β= 0.341, P=0.003). Social networks mediate responses to caregiving experiences among different care provider types and are vital intervention targets, especially for spousal caregivers. Our results can serve as references for identifying caregivers for clinical intervention.

